# Investigating the Associations between Ethnic Networks, Community Social Capital, and Physical Health among Marriage Migrants in Korea

**DOI:** 10.3390/ijerph15010147

**Published:** 2018-01-17

**Authors:** Harris Hyun-soo Kim

**Affiliations:** Department of Sociology, Ewha Womans University, Ewhayeodae-gil, Seodaemun-gu, Seoul 120-750, Korea; harrishkim@ewha.ac.kr

**Keywords:** ethnic ties, community social capital, bonding and bridging networks, self-rated health, marriage migrants

## Abstract

This study examines factors associated with the physical health of Korea’s growing immigrant population. Specifically, it focuses on the associations between ethnic networks, community social capital, and self-rated health (SRH) among female marriage migrants. For empirical testing, secondary analysis of a large nationally representative sample (NSMF 2009) is conducted. Given the clustered data structure (individuals nested in communities), a series of two-level random intercepts and slopes models are fitted to probe the relationships between SRH and interpersonal (bonding and bridging) networks among foreign-born wives in Korea. In addition to direct effects, cross-level interaction effects are investigated using hierarchical linear modeling. While adjusting for confounders, bridging (inter-ethnic) networks are significantly linked with better health. Bonding (co-ethnic) networks, to the contrary, are negatively associated with immigrant health. Net of individual-level covariates, living in a commuijnity with more aggregate bridging social capital is positively linked with health. Community-level bonding social capital, however, is not a significant predictor. Lastly, two cross-level interaction terms are found. First, the positive relationship between bridging network and health is stronger in residential contexts with more aggregate bridging social capital. Second, it is weaker in communities with more aggregate bonding social capital.

## 1. Background

Increasingly, public health scientists have focused on the “social determinants of health” [1]. In particular, social capital has emerged as one such factor underlying health inequality not only among individuals but also between groups, communities and even nations [2,3,4,5,6,7]. Disagreements over its precise definition notwithstanding, social capital denotes resources (information, emotional support, instrumental assistance, etc.) that exist in and are accessed through interpersonal relationships, group memberships and communal affiliations [8,9,10,11,12]. Empirical evidence abounds across multiple disciplines illustrating how access to social capital can have salubrious results, while its absence can lead to deleterious outcomes [5,13,14,15,16].

Whether “social capital is good for health” has figured prominently in many social epidemiological studies, with a particular emphasis on native (i.e., non-immigrant) populations in North American and European countries [17,18,19]. Today, in light of the increasing cross-border movements of people, understanding and improving immigrant wellbeing has become an urgent task from the global public health perspective [20]. One unique aspect of contemporary international migration is that it is no longer a “South-to-North” phenomenon. Rather, migratory flows have become much more multidirectional, transforming once labor-exporting countries into popular destinations for would-be migrants. Among them is Republic of Korea (hereafter Korea), which has experienced a “migration transition” in recent years [21]. Between 1990 and 2007, the number of foreign migrant workers in Korea rose dramatically from just 50,000 to over one million immigrants, a twenty-fold increase [22,23].

Along with this migratory inflow has been the rising presence of foreign-born wives, largely due to the shrinking pool of marriageable women in Korea’s countryside [24,25]. In 1990, there were only 4710 officially recorded biracial marriages in the country, which more than doubled to 12,319 a decade later, according to government data (Statistics Korea). By 2009, the year in which the national survey analyzed for this study was conducted, biracial unions accounted for 10.8% of all newly registered marriages [24]. In terms of the origin countries, according to the dataset analyzed for this study (National Survey of Multicultural Families 2009) which sought to survey all marriage migrants in Korea at the time, ethnic Koreans from China (36%) constitute the largest group, followed by Vietnamese (26%), Han Chinese (13%), and Japanese (5%).

Since the 1990s, female marriage migrants primarily from Southeast Asia, in particular, have arrived in Korea in huge numbers. In response to the influx, in 2006 the Korean government implemented the Plan for Supporting Social Integration of Women Marriage Immigrants and Families, whose main goal was to assist the adaptive process of foreign-born spouses of native Korean men. This plan provided the basis for the subsequent 2008 Multicultural Family Support Act (MFSA), which officially institutionalized state support for Korea’s growing biracial families and their children. Under this law, numerous programs and campaigns were developed with the intention of assimilating “multicultural families” into mainstream Korean society. Multiple centers have been established throughout the country to help implement immigrant-friendly policies. The welfare of foreign brides has been precarious, however, as they struggle to become assimilated.

Despite the profound demographic shifts, however, there is relatively little research on the health status of immigrants (e.g., foreign wives) in Korea. Few studies that address this topic primarily emphasize “acculturation” related stresses and problems [26,27,28,29,30]. How social capital is related to immigrant wellbeing, therefore, remains elusive. In light of this empirical gap, one main objective of this paper is to probe the associations between social capital and self-rated health (SRH) among female marriage migrants in Korea. Currently, there is an ongoing debate on how to properly conceptualize social capital. A growing number of scholars have suggested that when it comes to explaining health outcomes, social capital should be seen as resources that are possessed by individuals as well as characteristic features of broader environments (e.g., neighborhood, community) in which they are embedded [31,32,33,34,35]. A critical question is whether the effect of contextual-level social capital exists, after adjusting for individual-level social capital (compositional effects) and other covariates. Another issue of interest is whether the purported health-network linkage varies across contextual units such as residential communities [36,37]. In recognition of this key theoretical agenda, this paper seeks to contribute to the literature by using hierarchical linear models to measure and analyze the impact of social capital at two distinct levels, individual and community.

With respect to immigrants and the role of social capital in socioeconomic adaptation, prior studies have distinguished between “bonding” and “bridging” types [38,39]. The former refers to networks among similar (homogeneous) actors, while the latter denotes networks among dissimilar (heterogeneous) actors [32,39,40]. To contextualize these concepts for the purposes of this study, immigrant bonding network captures social resources available via connections to co-ethnic members. The bridging network measures those available through ties to interethnic (native Korean) members [41,42,43]. Extant research indicates that the bridging type provides instrumental value for immigrants in terms of formal sector employment, higher income, job stability, etc. Bonding social capital, on the other hand, may create negative labor market consequences by excluding immigrants from the benefits of cross-cutting ties with members of mainstream society [44,45,46,47].

While this conceptual dichotomy has become recognized in the social epidemiology literature concerning general populations, it has not been systematically applied to the study of immigrant health. Due to data limitations, the vast majority of previous research does not differentiate between bridging (i.e., out-group) and bonding (i.e., in-group) forms of social capital, which have divergent implications and consequences [33,48,49]. Using multilevel analysis, the current study thus sheds light on the ways in which individual social capital, community characteristics, and health are intertwined in the context of Korea’s immigrant community of foreign-born spouses, a topic that has escaped systematic scholarly attention.

Data analysis is informed by the following research questions: What is the nature of the relationship between the two aforementioned types of immigrant social capital and self-rated health (SRH)? More specifically, does bridging network enhance immigrant health, while bonding network diminishes it? And net of individual network ties, does living in a community with more bridging social capital lead to better health, whereas residence in a community with more bonding social capital diminishes it? Finally, how do neighborhood characteristics (aggregate-level social capital) moderate the associations between SRH and the two individual-level network measures? Using the administrative (census) and geo-coded micro survey data, this study addresses these pertinent, yet neglected, queries. A scoping review of the literature (academic articles published in English during the last 15 years) reveals that the current research is one of the very few attempts to systematically probe the nexus between immigrant SRH and the aforementioned dual conceptions of social capital using population-based data in a multilevel statistical framework.

## 2. Methods

### 2.1. Study Population

Data for this study are drawn from the National Survey of Multicultural Families (NSMF) 2009, a government-funded project supervised by the Korean Ministry of Gender, Equality & Family (MOGEF) [50]. NSMF (2009) is the first and the largest of its kind ever undertaken in the country. The data collection was carried out by Statistics Korea. The study population consisted of all foreign-born spouses living in Korea at the time of the survey, approximately 131,000 individuals. The survey was designed to interview the entire population. The response rate was 55.9%, resulting in a sample size of 73,669. Weights are provided by NSMF (2009) to account for the probability of selection based on multiple individual and residential characteristics. The study protocol was originally reviewed and approved by the Institutional Review Board affiliated with the Korea Institute for Health and Social Affairs (KIHASA). Face-to-face interviews were conducted with spouses of foreign origin, both men and women, located throughout the country’s 7 metropolitan cities and 9 provinces. The analysis is based on a subsample of immigrant wives who make up the vast majority (over 90%) of the dataset. After excluding male respondents (husbands) and divorced female respondents, along with the listwise deletion of cases with missing values, the final sample contains 67,143 foreign wives nested in 212 residential clusters or primary sampling units (PSUs).

### 2.2. Measures

The outcome variable (*SRH*) is based on individual responses to the subjective or self-rated health question, which has been shown to be a reliable predictor of morbidity and mortality [49]. A systematic review on social capital and health inequalities also cites SRH to be the most commonly used measure in the related field of research [16]. Measures for bonding and bridging *network* (at the individual level) and *social capital* (at the community level) are based on survey items that assessed levels of social connections and activities of immigrants. For each of the items, the respondents were asked to check the appropriate box corresponding to the ethnic identity of their social contacts (i.e., “Someone from my own country” or “Native Korean”). Based on this information, variables *Bonding Network* and *Bridging Network* are created, ranging in value from 0 (if the respondent did not check any of the boxes) to 3 (if all three were checked).

In addition to individual-level effects, contextual effects are examined. To that end, consistent with a standard practice in the literature, individual responses are averaged across community clusters to create three aggregate measures: *Social Activities*, *Bonding SC*, and *Bridging SC*. Census data were used to gauge five additional community-level characteristics. Information for these variables was obtained from the Korean Statistical Information Service (KOSIS), the government body in charge of collecting, managing, and disseminating official statistics (more details can be found at http://kosis.kr/eng). Using the geocoding information available in the NSMF (2009), the respondent-level data were merged with the KOSIS aggregate statistics to create a 2-level hierarchically nested dataset. Finally, to account for confounding effects, a number of sociodemographic and human capital controls are included in the analysis to provide a conservative examination of the associations between bonding and bridging networks and self-rated health. Details of coding scheme for and definition of all the variables are reported in Table 1. Table 2 summarizes the descriptive statistics.

### 2.3. Analytic Approach

The NSMF (2009) data are nested, i.e., the survey respondents were sampled across different geographically bounded administrative units. In other words, the data structure violates the basic independence assumption required in running conventional (OLS) regression models. This creates the problem of underestimating standard errors, thereby increasing the probability of committing the Type I error. To address this methodological issue and also to simultaneously examine individual- and contextual-level effects, multilevel analysis is performed. Since the dependent variable (*SRH*) is dichotomous, hierarchical generalized linear models (with a Bernoulli logit function) are estimated. Cases were weighted to account for the unequal probability of selection, and all inferential statistics were computed using robust standard errors to address the issue of non-normal distribution of data [51,52]. To deal with the collinearity problem, all non-dichotomous level-1 variables are group-mean centered, and all level-2 predictors in the models are grand-mean centered. A series of two-level random intercepts and slopes models were fitted using the latest version (7.1) of HLM (Hierarchical Linear Modeling) for Windows [53].

## 3. Results

Statistical results from estimating two-level logit models are reported in Table 3 and Table 4. Table 3 presents models containing individual-level covariates only. Table 4 contains results consisting of contextual-level direct and indirect (cross-level interaction) effects. Examination of the variance component from the unconditional model (Model 1 in Table 3) reveals significant between-cluster variability, validating the use of multilevel analysis (*τ*_00_ = 0.025, χ^2^ = 569.67, *p* < 0.001). Model 2 in Table 3 shows the control variables only, which, except for the number of children and the employment status, are all significantly associated with self-rated health. 

Model 3 incorporates the main predictor variables, bonding and bridging network measures. Adjusting for confounders, they are both significantly related to immigrant SRH, albeit in opposite directions. Bonding ties are negatively associated with health (OR = 0.96, *p* < 0.001), while bridging relations are positively related to it (OR = 1.38, *p* < 0.001). Inclusion of these two variables does not significantly alter the magnitudes of other covariates. One noticeable exception is the parameter estimate for *Social Activities*, which becomes insignificant suggesting that the effect of this variable is mediated through bonding and bridging networks of immigrants. In Model 4, the slopes for *Bonding Network* and *Bridging Network* are allowed to vary across the contextual units. The results are consistent: interaction with more co-ethnic contacts has a negative effect on SRH. By contrast, interaction with more inter-ethnic (native Korean) contacts has a positive impact.

To check for possible contextual effects, community-level variables are introduced as shown in Table 4. Model 1 consists of control variables only at the contextual level, while adjusting for all the individual-level covariates. Coefficients for the two main predictor variables (*Bonding Network* and *Bridging Network*) remain robust, even after holding constant the six community-level characteristics. Model 2 incorporates aggregate-level measures for bonding and bridging social capital. One significant result is that being embedded in a residential context with a higher proportion of bridging social capital is positively associated with subjective physical wellbeing (OR = 1.50, *p* < 0.01). Bonding social capital, on the other hand, is not a significant predictor of SRH. Finally, cross-level interaction effects are investigated. The association between bonding network and health is not contingent upon any of the community-level characteristics (models not shown). Two significant findings do emerge, however, with respect to bridging network. Model 3 tests if and the extent to which the protective relationship between the individual-level bridging network and SRH varies according to the community-level bridging social capital. Model 4 examines whether the same linkage varies across the community-level bonding social capital. Both interaction terms are significant. According to findings, the bridging network-health connection is stronger in communities with more aggregate bridging social capital (OR = 1.34, *p* < 0.01). To the contrary, it is weaker in residential clusters with more aggregate bonding social capital (OR = 0.75, *p* < 0.01).

## 4. Discussion

Based on the secondary analysis of a probability sample of Korea’s immigrant wives, the current research probed the associations between physical (self-rated) health and two types of interpersonal networks: bonding and bridging. It also investigated direct contextual-level and cross-level interaction effects. While adjusting for a host of background variables, the estimated multilevel models revealed that more bridging ties (with native-born members) leads to better immigrant health. Net of individual-level network measures, residence in a community with more aggregate bridging social capital was also positively related to health. In addition, the relationship between bridging network and health was stronger in residential contexts with higher average bridging social capital. And the reverse was true for communities characterized by more network connections among co-ethnic members (fellow immigrants). 

Why do the two types of networks, bonding and bridging, have such opposing implications for immigrant health at the individual level? What does the contextual effect of bridging social capital imply? And how do we account for the effect heterogeneity illustrated by the two cross-level interaction terms? In the general scholarship on immigration, bridging networks are seen more or less synonymous with interethnic or heterogeneous ties, while bonding networks are defined primarily in terms of co-ethnic or homogeneous ties. For many immigrants, including those analyzed in this study, it is natural to seek the presence of others with whom they share a common ethnic background and ancestry during the process of acculturation and adaptation. The existence of ethnic enclaves, such as Chinatown and similar others, in host societies is a clear testament to this seemingly ubiquitous tendency. After all, given the typical language and other cultural and institutional barriers, immigrants naturally seek and find support (social capital) from other people of foreign origin. Therein, however, lies the irony: all things equal, those who maintain more co-ethnic, or bonding, ties are penalized in terms of poor health. In contrast, those who have more interethnic, or bridging, ties benefit from better health. This is true even after adjusting for multiple factors known to correlate with immigrant health, including income and human capital measures such as education, length of stay and language skills.

These divergent outcomes can partly be attributed to the fact that interethnic contacts (native Koreans), in comparison with their foreign-born counterparts, are better able to offer the kinds of information and other resources necessary in maintaining and promoting physical wellbeing. Fellow immigrants, by definition, are more likely to be embedded in an ethnic enclave, detached from the mainstream Korean society, where access to relevant and timely social capital might be limited, if not unavailable. A Vietnamese wife, for example, who is well-connected to Korean friends is better positioned to, say, learn about the latest medical services and treatments, get referred to a qualified doctor, and acquire healthy dietary habits, among other benefits. An ethnic Chinese wife surrounded mainly by fellow Chinese immigrants, on the other hand, may face disadvantages in terms of gaining access to such knowledge and support. 

In short, it is only the bridging (cross-cutting) networks that play a protective role when it comes to the health status of female marriage migrants. Moreover, there is a kind of spillover effect where being surrounded by co-residents who have more interethnic ties (connections to Koreans) can enhance individual health, irrespective of one’s own level of social connections. In other words, geography or place matters. The estimated cross-level interaction terms corroborate these findings concerning the main or direct effects. More specifically, the positive role of bridging network is stronger in communities where immigrant residents are more connected with native Koreans but weaker in places where they are better connected with fellow foreign-born members. This effect heterogeneity suggests that the individual network-health relationship is not constant but contingent upon community-level social capital. In sum, this research highlights that, in addition to interpersonal networks, the degrees of ethnic composition and interaction that characterize the broader community environment in which immigrants are embedded have significant health-related implications for them.

There is a substantial literature on “neighborhood effects”, which contends that communities and even societies equipped with more collective social capital are conducive to better health, both mental and physical, for their members [18,32,36]. This study adds a novel, if not anomalous, insight to that scholarship by showing that “more” is not necessarily “better”. In fact, being densely connected to fellow immigrants produces a liability in terms of poorer subjective health. Prior research on contextual-level social capital using primarily non-immigrant or native-population data has not fully recognized this empirical fact. One of the main findings of this research is that while the *quantity* of social embeddedness is a critical factor enhancing individual health status, its *qualitative* dimension may be just as, if not more, important. A number of key questions emerge in light of this study. Among general population groups, what is the relationship between network types and individual health? Are the results discussed above replicable using non-immigrant data? For a native US citizen, for instance, how does bridging versus bonding ties influence his or her health behaviors and outcomes? And what are the health implications of living in a neighborhood that is characterized by dense bridging versus bonding networks? Given that many host societies consist of neighborhood units that are demarcated along racial and ethnic lines, pursuing these questions for immigrant-origin and native-born members may yield consequential results with profound health policy implications.

This study has some limitations. First of all, as is the case with most large-scale surveys, NSMF (2009) consists of cross-sectional data. As a result, the findings reported and discussed herein are correlational, not causal. This is a common shortcoming in the interdisciplinary research on the social determinants of health. Social capital (bonding and bridging networks) may have an impact on one’s physical wellbeing. Yet, reverse causation cannot be ruled out: that is, health status can influence the type and degree of social interaction and ultimately shape access to network-mediated resources. Collecting longitudinal data is necessary to minimize the problem of endogeneity and make it possible to draw accurate causal inference. The current study used self-rated health as the outcome variable. Employing objective measures of health conditions and symptoms would provide additional evidence concerning the main relationship under investigation. Another issue of data limitation has to do with the main predictor variables. In the NSMF (2009) questionnaire, respondents were asked about their respective social life in a limited number of domains. Expanding the survey items to include greater details of social activities would shed more light on the health-network linkage. Similar to other surveys, NSMF (2009) also did not gather specific information concerning how and the extent to which respondents rely on social networks for health purposes. An interpersonal tie may or may not channel information and support that are relevant and helpful. This study assumed that social interaction with inter-ethnic (co-ethnic) contacts necessarily leads to health-related (dis)advantages. More concrete variables, which measure the specific contents of network relations (e.g., giving and receiving health-related material assistance and emotional support) are needed to flesh out the complex ways in which different kinds of network connections can promote, or hinder, physical wellbeing. Moreover, the contextual (cluster) variable provided in NSMF (2009) may not properly capture the concept of “residential community”, given its relatively large average population size. Properly testing whether there is indeed a neighborhood effect on individual health outcomes calls for data consisting of smaller-level contextual units (e.g., census tracts or blocks). Lastly, additional studies would be needed to see whether the findings reported above on immigrants apply to non-immigrants as well, for which cross-national survey design is required.

## 5. Conclusions

The contingent relationships found in this study point to a complex interplay between immigrants’ individual networks, contextual social capital, and health inequality. Conventional knowledge has it that being socially well-connected has health benefits, at both individual and community levels. According to the aforementioned main findings, that actually depends on the type of connections. That is, the identities of one’s network contacts and co-residents are of critical importance. For the empirical subjects analyzed in the current study, there is an apparent paradox: being closely connected to other immigrants of the same ethnicity can be deleterious while ties to dissimilar others (members of a different ethnicity) can be salubrious. This paradoxical complexity operates at the contextual level as well. That is, living in a residential area with more aggregate bridging, but not bonding, social capital is associated with health benefits. Moreover, the positive relationship between bridging network and health is stronger (weaker) in residential contexts with higher average bridging (bonding) social capital. In the extant literature, very few studies have examined the role of community bonding/bridging social capital on individual SRH [32,33,34]. And virtually no study has systematically explored whether and how co-ethnic and inter-ethnic ties are differentially related to immigrant SRH. The current research advances the scholarship by offering new evidence based on a hitherto understudied empirical case: Korea’s increasing population of female marriage migrants. Future studies should take a comparative perspective in analyzing the network-health nexus in both immigrant and non-immigrant contexts, which could shed new light on how, when and why social connections and related resources matter for physical and mental wellbeing. 

## Figures and Tables

**Table 1:** Summary of variable definition and coding scheme.

Outcome Measure *SRH*	Respondent’s (R’s) self-rated health scores dichotomized, originally coded on a 5-point scale (e.g., 5 = very healthy, 1 = very unhealthy) 1 = very healthy and healthy; 0 otherwise
(Individual-level)	
*Age*	R’s age at the time of the survey in 2009
*SWB* *Income* *Marital Satisfaction*	R’s assessment of how happy she is with her life in general, coded on a 11-point ladder-type scale, ranging in value from 0 (lowest) to 10 (highest) R’s household income (logged) How satisfied are you with your marriage? (Answers dichotomized such that 1 = “very satisfied” and “satisfied”; 0 otherwise)
*Children*	“How many children do you have?”
*Education*	R’s highest formal educational attainment in her native country prior to moving to Korea (e.g., 1 = no education, 2 = elementary school, 5 = high school, 6 = graduate school)
*Residency*	“What is the total amount of time (in months) you have lived in Korea?” Answers log-transformed due to right-tailed skewed distribution
*Language*	A scale variable combining answers to the following three dimensions of language proficiency: “How well do you speak/read/write Korean?” Original answers coded on a 5-point scale (1 = very poor, 3 = average, 5 = excellent) are summed and averaged. Cronbach’s alpha = 0.94
*Korean Chinese*	R’s ethnicity (1 if she is an ethnic Korean from China)
*Chinese* *Vietnamese*	R’s ethnicity (1 if she is of Chinese descent) R’s ethnicity (1 if she is of Vietnamese descent)
*Employed*	“What is your working status?” (1 = working full-time; 0 otherwise, including part-time and enclave employment)
*Social Activities* *Bonding Network* *Bridging Network*	“During the last year, how often have you participated in the following social meetings and informal gatherings?” Four categories include those related to (1) my family/relatives; (2) my spouse’s family/relatives; (3) co-ethnic friends; and (4) local/residential (e.g., 1 = more than twice a week, 3 = once or twice a month, 5 = once a year, 6 = never) Answers added and log-transformed *Survey items used to gauge ethnic social capital at individual and contextual levels:* “Who do you consult concerning private matters or personal issues? With whom do you spend time together doing hobbies? Whose weddings, funerals and birthdays have you attended? Respondents asked to check the appropriate answer that applies to each of the three questions (0 = none, 1 = co-ethnic member, 2 = native Korean). Total number of co-ethnic contacts, as defined above (Cronbach’s alpha = 0.74) Total number of inter-ethnic contacts, as defined above Cronbach’s alpha = 0.73)
(Community-level)*Population Size* *Elderly Rate* *College Graduates* *Immigrants* *Commercialization* *Social Activities* *Bonding SC* *Bridging SC*	Logged population size Number of older adults aged 65 and over per 1000 residents Percentage of residents with tertiary education Proportion of foreign-born residents, per 100 natives Number of businesses, per 1000 residents Community-level mean for the number of social activities Community-level mean for the number of boxes checked for “co-ethnic member” Community-level mean for the number of boxes checked for “native Korean”

Source: National Survey of Multicultural Families (2009) [50].

**Table 2:** Descriptive statistics.

Variable	Mean/Proportion	S.D.	Min.	Max.
Outcome Measure				
*SRH*	0.51	-	0	1
(Level-1 *n* = 67,143)				
*SWB*	3.72	0.92	1	5
*Employed*	36%	-	0	1
*Marital Satisfaction*	72%	-	0	1
*Age*	33.37	9.97	18	92
*Children*	0.94	0.86	0	3
*Education*	3.73	0.94	1	6
*Income*	2.13	1.10	0	8
*Residency*	3.80	0.94	0	6.65
*Language*	3.24	1.08	1	5
*Korean-Chinese*	36%	-	0	1
*Chinese*	13%	-	0	1
*Vietnamese*	28%	-	0	1
*Social Activities*	1.71	0.78	0	3.26
*Bonding Network*	1.17	1.15	0	3
*Bridging Network*	1.57	1.16	0	3
(Level-2 *n* = 212)				
*Elderly Rate*	112.53	76.92	23.10	399.90
*College Graduates*	17.58	9.41	4.60	59.30
*Immigrants*	2	1.45	0.30	10.60
*Commercialization*	72.28	37.76	39.80	456.50
*Population Size (ln)*	6.59	2.18	3	10.28
*Social Activities*	1.71	0.12	1.20	1.98
*Bonding SC*	1.17	0.14	0.86	1.74
*Bridging SC*	1.58	0.14	1.07	2.00

Source: National Survey of Multicultural Families (2009) [50].

**Table 3:** Multilevel models of the associations between SRH and social capital measures (Individual-level covariates only).

*Fixed Effects*	Model 1 OR (CI)		Model 2 OR (CI)		Model 3 OR (CI)		Model 4 OR (CI)	
Constant	1.086	(1.06–1.12) ***	1.049	(0.98–1.13)	1.106	(1.03–1.19) **	1.109	(1.03–1.19) **
Individual-level variables								
*SWB*			1.546	(1.51–1.59) ***	1.526	(1.48–1.57) ***	1.527	(1.49–1.57) ***
*Employed*			0.942	(0.88–1.01)	0.948	(0.88–1.02)	0.945	(0.88–1.02)
*Marital Satisfaction*			1.957	(1.85–2.07) ***	1.873	(1.77–1.98) ***	1.872	(1.77–1.98) ***
*Age*			0.969	(0.97–0.97) ***	0.969	(0.97–0.97) ***	0.969	(0.97–0.97) ***
*Children*			0.998	(0.96–1.03)	1.006	(0.97–1.04)	1.008	(0.97–1.05)
*Education*			1.038	(1.01–1.07) *	1.031	(1.00–1.07)	1.031	(1.00–1.07)
*Income*			1.152	(1.13–1.18) ***	1.151	(1.12–1.18) ***	1.150	(1.12–1.18) ***
*Residency*			0.856	(0.82–0.90) ***	0.841	(0.80–0.88) ***	0.841	(0.80–0.88) ***
*Language*			1.211	(1.18–1.25) ***	1.187	(1.15–1.22) ***	1.187	(1.15–1.22) ***
*Korean Chinese*			0.680	(0.64–0.73) ***	0.664	(0.62–0.71) ***	0.663	(0.62–0.71) ***
*Chinese*			0.759	(0.70–0.82) ***	0.749	(0.69–0.81) ***	0.748	(0.69–0.81) ***
*Vietnamese*			0.505	(0.47–0.55) ***	0.513	(0.47–0.56) ***	0.512	(0.47–0.56) ***
*Social Activities*			1.057	(1.02–1.10) **	1.024	(0.99–1.07)	1.024	(0.99–1.07)
*Bonding Network*					0.959	(0.94–0.98) ***	0.958	(0.94–0.98) ***
*Bridging Network*					1.138	(1.11–1.17) ***	1.143	(1.12–1.17) ***
*Random Effects*								
Intercept (u_0j_)	0.0252 ***		0.0221 ***		0.0243 ***		0.0242 ***	
*Bonding Network* (u_1j_)							0.0065 ***	
*Bridging Network* (u_2j_)							0.0078 ***	
Reliability*_intercept_*	0.594			0.459		0.462		0.461

Note: Findings are from unit-specific models with robust standard errors. Source: NSMF 2009. * *p* < 0.05; ** *p* < 0.01; *** *p* < 0.001.

**Table 4:** Multilevel models of the associations between SRH and social capital measures (Individual- and contextual-level covariates).

*Fixed Effects*	Model 1 OR (CI)		Model 2 OR (CI)		Model 3 OR (CI)		Model 4 OR (CI)	
Constant	1.093	(1.02–1.17) *	1.104	(1.03–1.19) **	1.105	(1.03–1.19) **	1.104	(1.03–1.19) **
Individual-level variables								
*SWB*	1.527	(1.49–1.57) ***	1.528	(1.49–1.57) ***	1.528	(1.49–1.57) ***	1.528	(1.49–1.57) ***
*Employed*	0.974	(0.90–1.05)	0.946	(0.87–1.02)	0.946	(0.87–1.03)	0.946	(0.87–1.02)
*Marital Satisfaction*	1.874	(1.77–1.98) ***	1.871	(1.77–1.98) ***	1.871	(1.77–1.98) ***	1.870	(1.77–1.98) ***
*Age*	0.969	(0.97–0.97) ***	0.969	(0.97–0.97) ***	0.969	(0.97–0.97) ***	0.969	(0.97–0.97) ***
*Children*	1.010	(0.97–1.05)	1.009	(0.97–1.05)	1.008	(0.97–1.05)	1.009	(0.97–1.05)
*Education*	1.031	(1.00–1.07)	1.032	(1.00–1.07)	1.032	(1.00–1.07)	1.032	(1.00–1.07)
*Income*	1.149	(1.12–1.18) ***	1.150	(1.12–1.18) ***	1.149	(1.12–1.18) ***	1.149	(1.12–1.18) ***
*Residency*	0.840	(0.80–0.88) ***	0.841	(0.80–0.88) ***	0.841	(0.80–0.88) ***	0.841	(0.80–0.88) ***
*Language*	1.184	(1.15–1.22) ***	1.186	(1.15–1.22) ***	1.186	(1.15–1.22) ***	1.185	(1.15–1.22) ***
*Korean Chinese*	0.670	(0.62–0.72) ***	0.667	(0.62–0.72) ***	0.666	(0.62–0.72) ***	0.667	(0.62–0.72) ***
*Chinese*	0.754	(0.70–0.82) ***	0.753	(0.70–0.82) ***	0.753	(0.70–0.81) ***	0.754	(0.70–0.82) ***
*Vietnamese*	0.510	(0.47–0.55) ***	0.513	(0.47–0.56) ***	0.513	(0.47–0.56) ***	0.513	(0.47–0.56) ***
*Social Activities*	1.025	(0.99–1.07)	1.025	(0.99–1.07)	1.025	(0.99–1.07)	1.024	(0.99–1.07)
*Bonding Network*	0.958	(0.94–0.98) **	0.958	(0.94–0.98) *	0.958	(0.94–0.98) *	0.959	(0.94–0.98) *
*Bridging Network*	1.143	(1.12–1.17) **	1.144	(1.12–1.17) *	1.142	(1.12–1.17) *	1.142	(1.12–1.17) *
Community-level variables								
*Elderly Rate*	1.000	(1.00–1.00)	1.000	(1.00–1.00)	1.000	(1.00–1.00)	1.000	(1.00–1.00)
*College Graduates*	1.004	(1.00–1.01)	1.004	(1.00–1.01)	1.004	(1.00–1.01)	1.004	(1.00–1.01)
*Immigrants*	1.015	(1.00–1.03)	1.014	(1.00–1.03)	1.014	(1.00–1.03)	1.014	(1.00–1.03)
*Commercialization*	1.000	(1.00–1.00)	1.000	(1.00–1.00) *	1.000	(1.00–1.00) *	1.000	(1.00–1.00) *
*Population Size (ln)*	0.993	(0.97–1.02)	0.997	(0.97–1.02)	0.997	(0.98–1.02)	0.996	(0.98–1.02)
*Social Activities*	1.619	(1.15–2.29) **	1.703	(1.15–2.29) **	1.692	(1.18–2.42) **	1.689	(1.18–2.42) **
*Bonding SC*			0.835	(0.56–1.25)	0.834	(0.56–1.25)	0.820	(0.55–1.23)
*Bridging SC*			1.502	(1.06–2.12) **	1.528	(1.08–2.16) **	1.508	(1.07–2.13) **
Cross-level Interactions								
*Bridging Network* X *ridging SC*					1.344	(1.11–1.62) **		
*Bridging Network* X *Bonding SC*							0.753	(0.63–0.90) **
*Random Effects*								
Intercept (u_0j_)	0.0221 ***		0.0169 ***		0.0170 ***		0.0168 ***	
*Bonding Network* (u_1j_)	0.0064 ***		0.0063 ***		0.0063 ***		0.0062 ***	
*Bridging Network* (u_2j_)	0.0077 ***		0.0077 ***		0.0068 ***		0.0068 ***	
Reliability*_intercept_*	0.441			0.383		0.384		0.382

Note: All models adjust for the individual-level covariates shown in Table 3. Source: NSMF 2009. * *p* < 0.05; ** *p* < 0.01; *** *p* < 0.001.
